# Using Artificial Intelligence to Revolutionise the Patient Care Pathway in Hip and Knee Arthroplasty (ARCHERY): Protocol for the Development of a Clinical Prediction Model

**DOI:** 10.2196/37092

**Published:** 2022-05-11

**Authors:** Luke Farrow, George Patrick Ashcroft, Mingjun Zhong, Lesley Anderson

**Affiliations:** 1 Institute of Applied Health Sciences University of Aberdeen Aberdeen United Kingdom; 2 Grampian Orthopaedics National Health Service Grampian Aberdeen United Kingdom

**Keywords:** orthopedics, prediction modelling, machine learning, artificial intelligence, imaging, hip, knee, arthroplasty, health care, patient care, arthritis

## Abstract

**Background:**

Hip and knee osteoarthritis is substantially prevalent worldwide, with large numbers of older adults undergoing joint replacement (arthroplasty) every year. A backlog of elective surgery due to the COVID-19 pandemic, and an aging population, has led to substantial issues with access to timely arthroplasty surgery. A potential method to improve the efficiency of arthroplasty services is by increasing the percentage of patients who are listed for surgery from primary care referrals. The use of artificial intelligence (AI) techniques, specifically machine learning, provides a potential unexplored solution to correctly and rapidly select suitable patients for arthroplasty surgery.

**Objective:**

This study has 2 objectives: (1) develop a cohort of patients with referrals by general practitioners regarding assessment of suitability for hip or knee replacement from National Health Service (NHS) Grampian data via the Grampian Data Safe Haven and (2) determine the demographic, clinical, and imaging characteristics that influence the selection of patients to undergo hip or knee arthroplasty, and develop a tested and validated patient-specific predictive model to guide arthroplasty referral pathways.

**Methods:**

The AI to Revolutionise the Patient Care Pathway in Hip and Knee Arthroplasty (ARCHERY) project will be delivered through 2 linked work packages conducted within the Grampian Data Safe Haven and Safe Haven Artificial Intelligence Platform. The data set will include a cohort of individuals aged ≥16 years with referrals for the consideration of elective primary hip or knee replacement from January 2015 to January 2022. Linked pseudo-anonymized NHS Grampian health care data will be acquired including patient demographics, medication records, laboratory data, theatre records, text from clinical letters, and radiological images and reports. Following the creation of the data set, machine learning techniques will be used to develop pattern classification and probabilistic prediction models based on radiological images. Supplemental demographic and clinical data will be used to improve the predictive capabilities of the models. The sample size is predicted to be approximately 2000 patients—a sufficient size for satisfactory assessment of the primary outcome. Cross-validation will be used for development, testing, and internal validation. Evaluation will be performed through standard techniques, such as the C statistic (area under curve) metric, calibration characteristics (Brier score), and a confusion matrix.

**Results:**

The study was funded by the Chief Scientist Office Scotland as part of a Clinical Research Fellowship that runs from August 2021 to August 2024. Approval from the North Node Privacy Advisory Committee was confirmed on October 13, 2021. Data collection started in May 2022, with the results expected to be published in the first quarter of 2024. ISRCTN registration has been completed.

**Conclusions:**

This project provides a first step toward delivering an automated solution for arthroplasty selection using routinely collected health care data. Following appropriate external validation and clinical testing, this project could substantially improve the proportion of referred patients that are selected to undergo surgery, with a subsequent reduction in waiting time for arthroplasty appointments.

**Trial Registration:**

ISRCTN Registry ISRCTN18398037; https://www.isrctn.com/ISRCTN18398037

**International Registered Report Identifier (IRRID):**

PRR1-10.2196/37092

## Introduction

### Background

Hip and knee osteoarthritis (OA) is a common and disabling condition that predominantly affects the older population. Within Scotland, the prevalence of OA among people aged >45 years is approximately 10% and 17% for hip and knee OA, respectively [1]. The associated pain and loss of function can be so severe that over 10% of patients report their health state as being “worse than death” [2]. Joint replacement or “arthroplasty” provides a very successful method of improving symptoms for those with end-stage OA [3]. However, there are substantial current waiting times for surgery across the United Kingdom, and it is often difficult for nonspecialists such as general practitioners (GPs) to determine who might benefit from operative intervention.

Programs such as Active Clinical Referral Triage have attempted to address this problem through senior clinical decision-making instead of triage. However, this is labor and time intensive, with hospital consultants challenged by the difficulty of accessing and integrating the wealth of available, routinely collected health care data. Artificial intelligence (AI) provides a new, exciting, and hitherto unexplored technology to address these problems, with the potential to rapidly, and correctly, prioritize patients for arthroplasty. The ability to include automated analysis of radiological images into predictive models is particularly unique and may be of great benefit in this clinical context. The automated stratification of patients based on routine electronic health data could streamline referrals and reduce waiting lists through improved system efficiency.

The potential of AI techniques to address the current challenges of arthroplasty service provision is particularly important given that the number of people with end-stage OA continues to rise alongside an aging population [4]. In 2019, over 15,000 hip and knee arthroplasty operations were performed in Scotland alone [5], with an anticipated increase of over 100% for both primary total knee arthroplasty and total hip arthroplasty in the United Kingdom from 2005 to 2030 [6]. As a result, the numbers of revision total knee arthroplasty and total hip arthroplasty are expected to rise by over 300% [6], placing even greater demand on arthroplasty services.

Current service demand levels are already placing a substantial strain on the National Health Service (NHS). Many trusts throughout the United Kingdom set arbitrary thresholds for patient-reported outcome measures (PROMs) or BMI to limit arthroplasty referrals, despite evidence that current thresholds are likely inappropriate and exclude patients who would benefit from surgery [7,8]. Furthermore, over 61% of patients still currently wait between 3-9 months for clinical review prior to surgery (unpublished Scottish national survey data), with significant increases expected in the future due to service disruption and the backlog associated with the COVID-19 pandemic [9].

In addition, there are major difficulties for nonspecialists in determining who may benefit from operative intervention, as the majority of patients lie in the middle of the clinical need curve. As a consequence, less than 50% of new patients seen in an arthroplasty clinic are deemed suitable for surgery (unpublished Scottish national survey data). The resulting prolonged waiting times for review and subsequent surgery then have a negative impact on patient health [10] and potentially create greater strain on primary care and physiotherapy services. These negative changes that occur during the preoperative period also significantly impact the potential health gains imparted following hip and knee arthroplasty surgery, with greater deterioration seen for those with longer wait times [11]. The subsequent lower levels of physical activity may have long-term, wide-ranging implications for general health and well-being [12], with a significant associated economic and societal impact [13].

These adverse consequences stem from an inability to correctly and rapidly select suitable patients for surgery; therefore, a new method of streamlining arthroplasty referrals to reduce wait times is urgently needed. In this paper, we will build upon the work done within Scotland through the Active Clinical Referral Triage toward providing a personalized and precise arthroplasty service. The use of AI and machine learning techniques within orthopedics is an expanding field and provides an excellent method of analyzing integrated data from multiple clinical information sources, such as those required to improve arthroplasty care pathways. By transforming the way that arthroplasty services are delivered, timely access to appropriate and cost-effective care could be achieved, thus reducing complications and improving long-term physical function with a substantial impact on key stakeholders.

### Primary Objectives

1. Develop a cohort of patients with referrals by GPs regarding assessment of suitability for hip or knee replacement, and collect laboratory, clinical, and imaging data from NHS Grampian via the Grampian Data Safe Haven (DaSH).
2. Determine the demographic, clinical, and imaging characteristics that influence the selection of patients to undergo hip or knee arthroplasty, and develop a tested and validated patient-specific predictive model to guide arthroplasty referral pathways.

## Methods

The AI to Revolutionise the Patient Care Pathway in Hip and Knee Arthroplasty (ARCHERY) project will be conducted through 2 linked work packages designed to deliver on the project objectives.

### Work Package 1: The Definition of a Grampian Regional Data Source and the Establishment and Validation of a Linked Orthopedic Health Care Data Set Using Routinely Collected Data

The first work package will use ready to access local regional data from NHS Grampian that combines routine administrative data systems with enriched local data. Similar linked data sets have been used extensively by the team at the Aberdeen Centre for Health Data Science, within which the candidate will be hosted. Techniques for data access and processing are described in detail later in the protocol. Patient demographic information (Standard Morbidity Record 01 [SMR01]), medication records (prescribing information system), laboratory data (Apex hematology and biochemistry), COVID-19 data, theatre records (Centricity Opera) and PROMs (local PROMs database) will be used to develop the core algorithms using combinations of the relevant clinical codes (eg, International Classification of Disease 10th Revision [ICD-10] or Office of Population Censuses and Surveys Classification of Interventions and Procedures version 4 [OPCS-4]). SMR01 and theatre records (Centricity Opera) will provide the main resource for identifying joint replacement through the relevant ICD-10 codes. Unstructured (eg, free text) information in clinical letters and radiology image data will be used to validate and enhance these detailed characterizations. Risk factors and outcome measure algorithms will also be developed and validated against electronic clinical records.

Clinical knowledge of the key parameters involved in surgeon decision-making regarding patient selection for arthroplasty operations, as well as a planned systematic review, will aid variable selection. Given the standardization of referrals through the national Scottish Care Information Gateway system and the widespread similarities in approach to joint replacement selection throughout the United Kingdom, the use of Grampian regional data should produce a model that is widely applicable. Furthermore, we will use the close links between the Industrial Centre for Artificial Intelligence Research in Digital Diagnostics (iCAIRD) sites in Aberdeen and Glasgow to ensure that all data sources used have relevance regarding potential future suitability for national application.

Subsequent operation and automation of these techniques will allow for systematic and reproducible approaches to characterize the key clinical features of the data that are relevant to orthopedics. The algorithms created will then be scaled and used to appropriately categorize and construct a linked data set, covering all relevant hospital episode data on patients with orthopedic referrals, that will be used in work package 2.

### Work Package 2: The Development of a Clinical Prediction Model to Help Guide Arthroplasty Selection

Using the cohort developed in work package 1, probabilistic and classification machine learning will be conducted through statistical analysis software programs (Rstudio [Rstudio PBC], Python, and Tensorflow [Google]) to predict whether or not a patient would be selected to undergo surgery based on preoperative clinical data (including imaging data and reports, clinical letters [through natural language processing], patient health care information, and PROMs). This will also include information about the patients’ likelihood of having a successful outcome, both in terms of functional improvement and avoidance of complications.

The machine learning models will use data from the predictive variables that were isolated from the preoperative routine health care data and described in work package 1. Pretrained convolutional neural networks (a type of machine learning categorized as deep learning) will be used for X-ray images to significantly increase generalizability, with the X-ray images providing the foundation for model creation. To facilitate model training, development, and internal validation, we will use k-fold cross-validation, allowing all data to be used for testing and internal validation purposes without sample attrition.

### Model Output

The machine learning model will create 2 types of output for the primary outcome–a classification model, where the output is a discrete binary selection, and a probabilistic model. This will provide different possibilities for future clinical application depending on key stakeholder input; the algorithm could either be used as an adjunct in the patient-GP clinical discussion and decision-making process regarding referral or as a postreferral triage system, where patients are stratified to see different orthopedic specialists based on their predicted suitability for surgery.

### Data Access and Processing

The project will be performed within the remit of the Grampian DaSH. This is located within the Aberdeen Centre for Health Data Science, which has considerable expertise and experience working across local, regional, and national systems for projects. Study outcomes of the proposed work will be shared through social media engagement, dissemination at relevant conferences, and publication.

Safe processing of NHS patient data using AI will be provided through collaboration with iCAIRD—a pan-Scotland collaboration of 15 partners, including the Universities of Aberdeen and Glasgow, and a world-class center of excellence for AI application to health care within Scotland. The project will use the iCAIRD Safe Haven Artificial Intelligence Platform located within the NHS domain to perform the machine learning analysis.

Although individual patient data cannot be shared, the metadata and information about the data access procedure and the data extraction methods will all be shared through online repositories (eg, GitHub).

### Recruitment

There will be no direct participant recruitment for this study. Unconsented, pseudo-anonymized data will be used. Inclusion criteria are defined as individuals aged ≥16 years who have been referred for the consideration of elective primary hip or knee replacement within NHS Grampian from January 2015 to January 2022. This cohort will be generated from Scottish Care Information–stored referral letters by identifying the patients who have been referred to elective orthopedic services within NHS Grampian and have the words “*hip” OR “knee” AND “arthritis” OR “pain”* (including stemming: eg, “osteoarthritis”) as free text information contained within the referral letter. This process will occur prior to data access by the study team. Exclusion criteria are defined as the following: individuals who have undergone revision hip or knee arthroplasty, arthroplasty at another site, or unicompartmental knee replacement; individuals who have undergone hip or knee replacement for trauma (hip fracture or distal femoral fracture); and individuals who have undergone operative management outside of NHS Grampian.

The research team will have no access to patient identifiers, such as name, date of birth, or Community Health Index. No identifiers will leave the NHS server. Identifiable data will be stored in DaSH on an NHS server with access restricted to NHS Health Intelligence analysts and approved DaSH analysts (with NHS Grampian honorary contracts).

Deidentified data (ie, data without patient identifiers) will be stored on a dedicated DaSH secure server with access to the data restricted to the named, approved DaSH staff who will prepare the data.

Access to the anonymized data will be via virtual private network, as per DaSH processes, and within a secure analytics platform using restricted access to high power computer clusters. The computers used will have limited access measures in place via usernames and passwords.

The project has a DaSH data management plan, which provides details of how the data will be transferred, managed, stored, and accessed (Multimedia Appendix 1).

Data linkage and management will be completed in DaSH using accredited procedures to collect, link, and pseudo-anonymize the electronic data and enable users to securely access the anonymized data and back up and recover data. The data linkage plan for the project is displayed in Multimedia Appendix 2.

The legal basis for processing unconsented personal data is covered by the condition set out in Article 6 (1) (e) of General Data Protection Regulation (GDPR) [14]: “[the condition for] processing [personal data is that it] is necessary for the performance of a task carried out in the public interest or in the exercise of official authority vested in the controller” (ie, processing is necessary for the University’s public interest task of conducting research).

The basis for the processing of sensitive personal data is outlined in Article 9 (2) (j) of GDPR [14]: “processing is necessary for archiving purposes in the public interest, scientific or historical research purposes or statistical purposes.”

### Sample Size

Sample size will be set by the number of patients that have sufficient data available; this is anticipated to be approximately 2000 patients from an initial screening of the records. It has previously been noted that over 90% accuracy can be achieved with a minimum of 500 samples when considering the development of convolutional neural networks, and accuracy increases with the size of the training cohort [15].

Using the guidance for binary clinical prediction models provided by Riley et al [16], we anticipate that this sample size will:

1. Estimate the overall outcome proportion with sufficient precision, which required a sample size of 384 with an outcome proportion of 0.5 and a margin of error of 0.05.
2. Target a small mean absolute prediction error, which required a sample size of 1890 with 20 candidate predictors, an outcome proportion of 0.5, and a mean absolute prediction error of 0.050.
3. Target a shrinkage factor of 0.9, which required a sample size of 800 with an *R*^2^ value of 0.2 [17], 20 candidate predictors, and a target *S* value of ≥0.9.
4. Target a small optimism of 0.05 in the apparent *R*^2^ value, which required a sample size of 36 with an anticipated *R*^2^ value of 0.2, an outcome proportion of 0.5, a maximum *R*^2^ value of 0.75, and 20 candidate predictors.

### Analysis

First, descriptive analyses of the generated cohort will be performed to evaluate the base characteristics, including the assessment of any missing data. The type and size of missing data will determine whether any formal data imputation techniques are required. If necessary, this will be done using multiple imputations by chained equations. Data fields with large volumes of missing data will be assessed using complete case analysis.

Univariable analyses will be performed to assess the association between the final included variables and outcomes. *t* tests and Mann-Whitney *U* tests will be used for parametric and nonparametric continuous data, respectively, and chi-square tests will be used for categorical data. In all tests, *P*<.05 will denote significance. The results will be reported with 95% CI.

Initial model development will consist of the use of raw imaging data alone, followed by sequential inclusion of variables deemed to be important from the univariable analyses and background clinical knowledge identified from key literature searching.

Multiple models will be generated, with evaluation performed through standard techniques such as the C statistic (area under curve) metric, calibration characteristics (Brier score), and a confusion matrix. The model that performs best against these domains will be chosen. A *κ* index will be used to compare the ability of the machine learning algorithms to detect severe arthritis requiring joint replacement against human observers using an observer-defined clinical categorization tool (Kellgren-Lawrence grading). Heat maps will be generated as part of the machine learning output to identify the areas of the included images that have contributed primarily to model classification using a technique called class activation mapping.

All analyses will be performed using R statistical software (R Foundation for Statistical Computing) as a base program, with additional input from other programs, such as Tensorflow and Python, as required.

### Ethics Approval and Conduct

Informed consent has not been sought, which is consistent with other studies performing retrospective review of pseudo-anonymized health data. The chief investigator and staff involved with this study will comply with the requirements of the GDPR [14] and Data Protection Act 2018 [18] with regards to the collection, storage, processing, and disclosure of personal information and will uphold the Act’s core principles.

The data will be managed by DaSH, the accredited regional safe haven for NHS Grampian, and in accordance with existing Caldicott and Research Ethics Committee approvals for DaSH. Linkage and anonymization will follow standard, approved, and accredited protocols and will be undertaken by DaSH staff.

The research team will undertake appropriate information governance training before gaining access to a dedicated DaSH research analytics platform.

Approval from the North Node Privacy Advisory Committee was confirmed on October 13, 2021 (Multimedia Appendix 3). This covers approvals from the following organizations: NHS Grampian Caldicott Guardian, Research Ethics Committee, NHS Grampian Research & Development, NHS Grampian Information Governance, and University of Aberdeen Research Governance.

All data, reports, and other records will be used in a manner designed to maintain participant confidentiality. The study will be conducted in accordance with the 1964 Helsinki declaration and its later amendments.

The chief investigator and staff involved with this study will not disclose or use the study data for any purpose, other than for the work described in this protocol and related documentation.

## Results

The study was funded by the Chief Scientist Office Scotland as part of a Clinical Research Fellowship that runs from August 2021 to August 2024.

Data collection started in March 2022, with the results expected to be published in the first quarter of 2024. ISRCTN registration has been completed (number 18398037).

The conduct and reporting of the study will be in-line with the Transparent Reporting of a Multivariable Prediction Model of Individual Prognosis or Diagnosis–Artificial Intelligence (TRIPOD-AI) statement, which is currently in the final stages of development [19].

## Discussion

### Expected Findings

We anticipate the development of an automated internally validated algorithm from routine health data that will provide a first step toward improving the selection of patients for referral to undergo hip and knee arthroplasty surgery.

This new pathway will help to maximize efficiency through improved quality of care and reduced administrative burden for clinical staff. This is particularly important in the context of the COVID-19 pandemic that has already seen substantial increased pressure placed on arthroplasty services.

### Comparisons to Prior Work

Other clinical research within this area is very limited. Many trusts throughout the United Kingdom set arbitrary thresholds for PROMs or BMI to limit arthroplasty referrals, despite evidence that current thresholds are likely inappropriate and exclude patients that would benefit from surgery.

### Strengths and Limitations

The strengths of this study include the availability of substantial volumes of relevant clinical data through the creation of a regional linked data set. The use of machine learning analysis and, particularly, the inclusion of clinical imaging data within a predictive model are novel and provide potential for improvement over pre-existing techniques. The development of automated systems using routine health data will ensure minimal additional burden and maximize cost-efficiency.

Limitations include the use of retrospective data collection, which limits the scope and type of information collected, as well as the need for further evaluation and development before use in clinical practice can be considered.

### Future Directions

Once developed, the prediction model will have to undergo external validation to ensure it retains its potential utility outside of NHS Grampian data. This will likely take place using the federated iCAIRD network, which would allow the deployment of the developed model algorithms to use NHS Greater Glasgow and Clyde data without the need for any data transfer out of the local safe haven environment.

The externally validated model will then be required to undergo assessment regarding the potential impact on clinical practice, ideally through a randomized controlled trial design. Input from key stakeholders, such as those in primary care, will be integral to ensuring that any model maximizes the potential for clinical impact through application at the correct stage of the patient journey.

Once in clinical practice, continual evaluation and further development of the model will be required to ensure that a high level of clinical applicability across populations is maintained.

## Appendix

Multimedia Appendix 1 Data management plan.

Multimedia Appendix 2 Data linkage plan.

Multimedia Appendix 3 North Node Privacy Advisory Committee Approval.

## Abbreviations

AI: artificial intelligence
ARCHERY: AI to Revolutionise the Patient Care Pathway in Hip and Knee Arthroplasty
DaSH: Data Safe Haven
GDPR: General Data Protection Regulation
GP: general practitioner
iCAIRD: Industrial Centre for Artificial Intelligence Research in Digital Diagnostics
ICD-10: International Classification of Disease 10th Revision
NHS: National Health Service
OA: osteoarthritis
OPCS-4: Office of Population Censuses and Surveys Classification of Interventions and Procedures version 4
PROM: patient-reported outcome measure
SMR01: Standard Morbidity Record 01
TRIPOD-AI: Transparent Reporting of a Multivariable Prediction Model of Individual Prognosis or Diagnosis–Artificial Intelligence
