# Supplementary material for: Using Artificial Intelligence to Revolutionise the Patient Care Pathway in Hip and Knee Arthroplasty (ARCHERY): Protocol for the Development of a Clinical Prediction Model
Source: JMIR Res Protoc. 2022 May 11;11(5):e37092. doi: 10.2196/37092 (PMC9133991; doi:10.2196/37092)
Supplement: Multimedia Appendix 1 [file resprot_v11i5e37092_app1.docx]

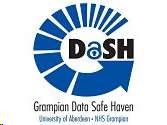
**Project Specific Data Management Plan (sDMP)**

**DaSH project number: 438 Project Type PI / Lead Supervisor**

Linkage Luke Farrow

**Project Title** ARCHERY Project: Ai to Revolutionise the patient Care pathway in Hip and knEe aRthroplastY

| **Approved Researchers requiring access to the de-identified data** | | | |  | | | | |
| --- | --- | --- | --- | --- | --- | --- | --- | --- |
| **Name** | | **Access** | | **Organisation** | | | | |
| Luke Farrow SHAIP (Remote)  George Ashcroft SHAIP (Remote)  Lesley Anderson SHAIP (Remote)  Mingjun Zhong SHAIP (Remote) | | | | NHS Grampian / University of Aberdeen  University of Aberdeen  University of Aberdeen  University of Aberdeen | | | | |
|  | | | |  | | | | |
| **Data Sources** | | | | |  | |  |  |
| **Dataset name** | | | | | **Updated periodically** | | **CHI**  **Indexing** | **Permanently De-identified** |
| PACS Reports | | | | | No (for all) | | No (for all) | No (for all) |
| PACS Images  Apex Haematology Grampian  Orthopaedic Patient Reported Outcome Measure (PROMs) database  Opera NHS Grampian  General/Acute Inpatient & Day Case dataset Grampian  TrakCare Hospital Electronic Health Records  Prescribing Information System for Grampian  Apex Biochemistry Grampian  COVID-19 Shielding List  Scottish Index of Multiple Deprivation (SIMD)  COVID-19 Testing  Outpatient Clinic Letter  Accident & Emergency NHS Grampian | | | | |  | |  |  |
| **Data Access and Archiving** | | | **De-identification of Data Sources** | | | **Data Sharing for Future Research** | | |
| **Archiving (Months)** | **Length of Study (Months)** | | **Details** | | | Please see General Data Management Plan or specify details if other rules apply **(see page 2 of this document for amendments)** | | |
| 60 36 | | | Pseudo-anonymised | | | **Suitable for sharing but with restriction** | | |

**Amendments to generic Data Management Plan (gDMP)**

Please note that for the current project (Artificial intelligence-supported early fracture diagnosis: SBRI competition), where we refer to Grampian Data Safe Haven or the DaSH analytics platform in the generic Data Management Plan (gDMP), we are referring to the Grampian Data Safe Haven Artificial Intelligence Platform (SHAIP). Please note the following amendments to the gDMP are also applicable for this project:

- Section 14 – The final de-identified dataset(s) for analysis will be stored and accessed on the secure Grampian Data Safe Haven Artificial Intelligence Platform (SHAIP).
- Section 15 – You are responsible for uploading your own Docker and ensuring that no malicious code is included, the VPN used to connect to SHAIP is shared with other researchers: please be respectful and do not upload docker images that are any larger than you need (see Canon’s Guide Section 2.5: Reducing Image Size for more information on this).
