# Supplementary material for: Using Artificial Intelligence to Revolutionise the Patient Care Pathway in Hip and Knee Arthroplasty (ARCHERY): Protocol for the Development of a Clinical Prediction Model
Source: JMIR Res Protoc. 2022 May 11;11(5):e37092. doi: 10.2196/37092 (PMC9133991; doi:10.2196/37092)
Supplement: Multimedia Appendix 2 [file resprot_v11i5e37092_app2.docx]

**Data Linkage Statistics**

| **External** | | |
| --- | --- | --- |
| **Dataset** | **Number of Rows** | **Notes** |
| **DS1:**  Identifiers and non-image data |  |  |
| **DS3:**  Images |  |  |

| **SHAIP: NHS Side** | | |
| --- | --- | --- |
| **Dataset** | **Number of Rows** | **Notes** |
| **DS2:**  Identifiers |  | **Identifiers from DS1 for extracting PACS**  **Identifiers from DS1 for extracting DaSH NHS Platform datasets** |
| **DS4:**  Non-image data |  | **Non-image data from DaSH NHS Platform datasets** |
| **DS5:** payload (DS1 &&DS 4) |  | **Full payload data Dataset to be anonymised** |
| **DS6:** Anonymised payload |  | **Anonymised payload data** |

| **SHAIP: University Side** | | |
| --- | --- | --- |
| **Dataset** | **Number of Rows** | **Notes** |
| **DS6:** Anonymised Payload Data |  | **DS6 from SHAIP NHS to be checked before release** |
|  |  |  |

**Data Specification**

Data will be extracted and processed as per the Project Data Definition Document

DLP Document History and Version Control

| **Version** | **Date** | **Author** | **Description** |
| --- | --- | --- | --- |
| 0.1 | 07-09-2020 | Jaroslaw Dymiter | DLP creation |
| 0.2 | 18-09-2020 | Jaroslaw Dymiter | Updates to flowchart & dataset numbers |
| 0.3 | 13-10-2020 | Jaroslaw Dymiter | Added PIS dataset |
| 0.4 | 19-10-2020 | Jaroslaw Dymiter | Moved PIS dataset |
| 0.6 | 12-04-2021 | Jaroslaw Dymiter | Added COVID datasets |
| 0.7 | 12/08/2021 | Artur Wozniak | Dataset re-positioning |
| 0.8 | 23/08/2021 | Adrian Martin | Dataset addition |
| 1.0 | 02/09/2021 | Jaroslaw Dymiter | Sign off |
| 1.0 | 21/02/2022 | Jaroslaw Dymiter | Added Radiology Reports dataset |
