# Supplementary material for: Using Artificial Intelligence to Revolutionise the Patient Care Pathway in Hip and Knee Arthroplasty (ARCHERY): Protocol for the Development of a Clinical Prediction Model
Source: JMIR Res Protoc. 2022 May 11;11(5):e37092. doi: 10.2196/37092 (PMC9133991; doi:10.2196/37092)
Supplement: Multimedia Appendix 3 [file resprot_v11i5e37092_app3.pdf]

13th October 2021

Lesley Anderson
Aberdeen Centre for Health Data Science,
1:170 Polwarth Building,
School of Medicine, Medical Science and Nutrition
University of Aberdeen
Foresterhill
Aberdeen
AB25 2ZD

Dear Professor Anderson,

CONFIRMATION OF APPROVAL

UK POLICY FRAMEWORK FOR HEALTH AND SOCIAL CARE RESEARCH: V3.3 7TH NOVEMBER 2017

**Chief Investigator:**	Lesley Anderson
**Title of Project:**	Ai to Revolutionise the patient Care pathway in Hip and knEe aRthroplastY (ARCHERY)
**Sponsor:**	The University of Aberdeen
**NNPAC Ref no:**	6/091/21

On behalf of the North Node Privacy Advisory Committee (NNPAC), I write to confirm that your application has been granted approval.

Approval is given to process data as specified in the approved application form. Approval is given until 04/08/2024.

As the Chief Investigator you have the responsibility, even when a task is delegated, to ensure that:

1. The research project complies with all applicable legal and local requirements **before** the study commences and thereafter.

2. The research team shall follow all relevant Standard Operating Procedures available on the University of Aberdeen and NHS Grampian Research Governance Website: http://www.abdn.ac.uk/clinicalresearchgovernance/sops

3. The project is registered on a public register prior to data access, and results reported within 12 months following study closure, unless agreed otherwise with the NNPAC committee.

4. The research follows the approved document set and approval from the NNPAC committee. This shall be obtained **prior** to submitting or implementing any amendment to

any aspect of your proposal, including purpose or method of processing, data or data variables being processed, study cohorts, individuals accessing and processing data, timescales, technology/infrastructure or any other relevant change.

5. Each member of the research team is qualified to undertake their role in the project, students are adequately supervised and the research will be performed to the principles of Good Clinical Practice (GCP).

6. The NNPAC committee and Sponsor of the research project is notified at the earliest opportunity of any long-term absence of the CI and agrees to the provisions made for the research project to continue. The Sponsor is to be notified immediately should the employment status of the CI change.

7. All records used, and data generated for the research project should be appropriately stored throughout and archived at the end of the research project.

8. NNPAC committee, funder and others as appropriate should be forwarded an annual progress report and on completion of the project, the End of Trial Declaration. NNPAC should be notified via nnpac@abdn.ac.uk.

9. Research findings, and where appropriate, data should be made available for critical review. Once established, findings must be made accessible through publication or other appropriate means. The final project report and any publication should be forwarded to NNPAC should be notified via nnpac@abdn.ac.uk.

10. The NNPAC committee and Sponsor is informed if important intellectual property is generated within the project; protection of such intellectual property must be considered prior to publication. NNPAC should be notified via nnpac@abdn.ac.uk.

I would like to remind you of the declaration you have made in your application form committing to undertakings in respect of information governance, confidentiality and data protection. You should be aware that once personal data (irrespective of de-identification or other controls applied) has been extracted and transferred to you, that you and the organisation you represent will become a data processor as defined by the data protection act, regardless of any other legal obligations that organisation may hold.

Yours sincerely,

NNPAC Representative

Approved Documents

Document	Version number	Date
Supplementary material 1 – Peer Review	NA	21.07.2021
Supplementary material 2 – Protocol	3	13.10.2021
Supplementary material 3 – DaSH438_DLP	1	02.09.2021
Supplementary material 4 – DaSH438_sDMP	1	23.08.2021
Supplementary material 11 – CAF_21_06- Offer of Award Letter	NA	NA
North Node Privacy Advisory Committee – Application Form	4	07.10.2021
CV and MRC for Lesley Anderson	NA	NA
CV and MRC for Luke Farrow	NA	NA
CV and MRC for Mingjun Zhong	NA	NA
